# Influence of APOE and ADAM10 Gene Polymorphisms on Cognitive Performance and Alzheimer's Disease Diagnosis in Older Adults from a Latin American Country

**DOI:** 10.1002/alz70855_103740

**Published:** 2025-12-23

**Authors:** Lucas Nogueira de Carvalho Pelegrini, Rebeca Carvalho Bom, Danilo Barroso de Sousa, Leticia Fernanda Palma, Ana Beatriz Aparecida Targas, Marina Mantellatto Grigoli, Sabrina Dorta de Oliveira, Patricia Regina Manzine, Cecilia Patricia Popolin, Marcia Regina Cominetti

**Affiliations:** ^1^ Federal University of São Carlos, São Carlos, São Paulo, Brazil; ^2^ Federal University of Sao Carlos, São Carlos, Sao Carlos, Brazil; ^3^ Federal University of São Carlos, São Carlos, SP, Brazil; ^4^ The Global Brain Health Institute, Trinity College Dublin, Dublin, Dublin, Ireland

## Abstract

**Background:**

Alzheimer's disease (AD) is a progressive neurodegenerative disorder influenced by modifiable and non‐modifiable risk factors. The APOE ε4 allele is a well‐established genetic risk factor for AD, while ADAM10, an important α‐secretase enzyme, has been implicated in amyloid precursor protein processing and is potential AD biomarker. However, the influence of ADAM10 polymorphisms on cognitive performance and AD diagnosis remains unclear. This study aimed to investigate whether APOE and ADAM10 polymorphisms influence cognitive performance in cognitively healthy older adults and people living with AD.

**Method:**

A total of 135 older adults, including cognitively unimpaired (CU) individuals and those diagnosed with AD, were assessed. Sociodemographic, clinical, and cognitive characteristics were collected through standardized questionnaires and neuropsychological tests, including the Addenbrooke's Cognitive Examination Revised (ACE‐R), Mini‐Mental State Examination (MMSE), and CERAD battery. APOE and ADAM10 polymorphisms were genotyped (qPCR), and their associations with cognitive performance and AD diagnosis were analyzed using t‐tests and logistic regression models, controlling for age and education. The significance level adopted was *p* <0,05 and all analyses were conducted in SPSS v. 29.0. All participants signed an an Informed Consent Form before data acquisition.

**Result:**

Participants with AD were older (*p* <0.001), had lower education levels (*p* = 0.029), and engaged in less physical activity (*p* <0.001) compared to cognitively healthy individuals. The AD group also reported greater memory complaints (*p* = 0.041), higher depressive symptoms (*p* <0.001), and worse functional status (*p* <0.001). Cognitive performance was significantly lower in the AD group across all neuropsychological measures (*p* <0.001). Logistic regression analysis indicated that carrying the APOE ε4 allele significantly increased the likelihood of an AD diagnosis (OR=10.11; *p* = 0.003; 95% CI [2.15‐47.6]). However, no significant associations were found between ADAM10 SNPs and AD diagnosis.

**Conclusion:**

This study reinforces the role of the APOE ε4 allele as a strong genetic risk factor for AD but found no evidence supporting an association between ADAM10 polymorphisms and AD diagnosis. These findings highlight the need for further research to elucidate the potential role of ADAM10 in cognitive aging and AD pathophysiology.

